# A Mechanistic Insight into the Molecular Mechanism of the Thermal Decomposition of Nitroalkyl Phosphates: MEDT Computational Study

**DOI:** 10.3390/ma18235312

**Published:** 2025-11-25

**Authors:** Przemysław Woliński, Ewa Dresler, Radomir Jasiński

**Affiliations:** 1 Cracow University of Technology, Department of Organic Chemistry and Technology, Warszawska 24, 31-155 Kraków, Poland; przemyslaw.wolinski@pk.edu.pl; 2 Łukasiewicz Research Network—Institute of Heavy Organic Synthesis “Blachownia”, Energetyków 9, 47-225 Kędzierzyn-Koźle, Poland; ewa.dresler@icso.lukasiewicz.gov.pl

**Keywords:** nitroalkenes, nitrocompounds, thermal elimination, β-elimination, mechanism, DFT

## Abstract

The kinetic aspects and molecular mechanism of the thermal decomposition of nitroalkyl phosphates were evaluated on the basis of DFT quantum-chemical calculations at the ωb97xd/6-311G(d,p) (PCM) level of theory. These reactions were found to proceed via a single-step mechanism with a six-membered transition state. This mechanism is similar to the mechanism of the elimination reaction of carboxylic acids from their esters. However, this is not a pericyclic mechanism. BET studies have shown that migration of hydrogen takes place before the breaking of the C-O bond. The effect of substituents on the nitroalkyl moiety of the ester on the reaction kinetics was also explored. Based on the obtained results, this mechanism can be proposed as general for a wider group of compounds.

## 1. Introduction

Organic compounds, including nitrovinyl molecular segment(s), play an important role in modern chemistry, chemical technology, biotechnology, pharmacy, and other related sciences. Conjugated nitroalkenes (CNAs) and conjugated nitrodienes (CNDs) are applied, inter alia, as pharmacokinetic agents [1], antibacterial drugs [2], SARS-CoV-2 inhibitors [3], anti-inflammatory agents [4], nicotinoid inhibitors [5], antifungal agents [6], anti-atherogenic agents [7], and many others [8,9,10,11]. The nitrovinyl molecular segment is strongly polarized and characterized by an important difference between local electrophilicities at potential reaction centers [12,13,14,15]. This fact determines its important application potential in the synthesis of five- [16,17,18,19] and six-membered heterocycles characterized by great bioactive potential [18,20,21,22]. The presence of the nitro group additionally offers great potential for the construction of other functional groups and molecular segments [23,24,25].

**Scheme 1:**
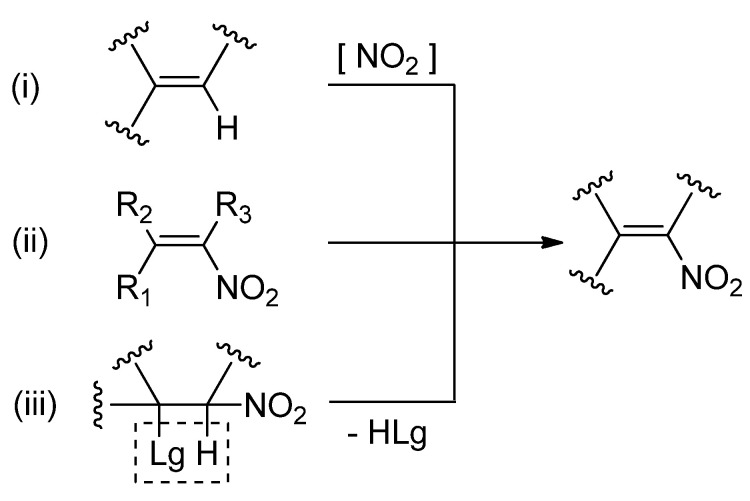
General strategies for the preparation of CNAs.

In the literature, many different methods for the preparation of conjugated nitroalkenes have been described (Scheme 1). Generally, three different approaches are considered: (i) nitration of unsaturated molecular segments [26,27], (ii) modification of other nitroalkenes [28,29], and (iii) formation of a double carbon-carbon bond in saturated nitrocompounds [30,31,32]. The first group of methods has very low selectivity due to the great affinity of the double bond to nitration agents. The second group of methods is possible only in the case of certain incidental structures. Only the last approach is universal within the acceptable range for the effective and selective preparation of different types of CNAs.

**Scheme 2:**
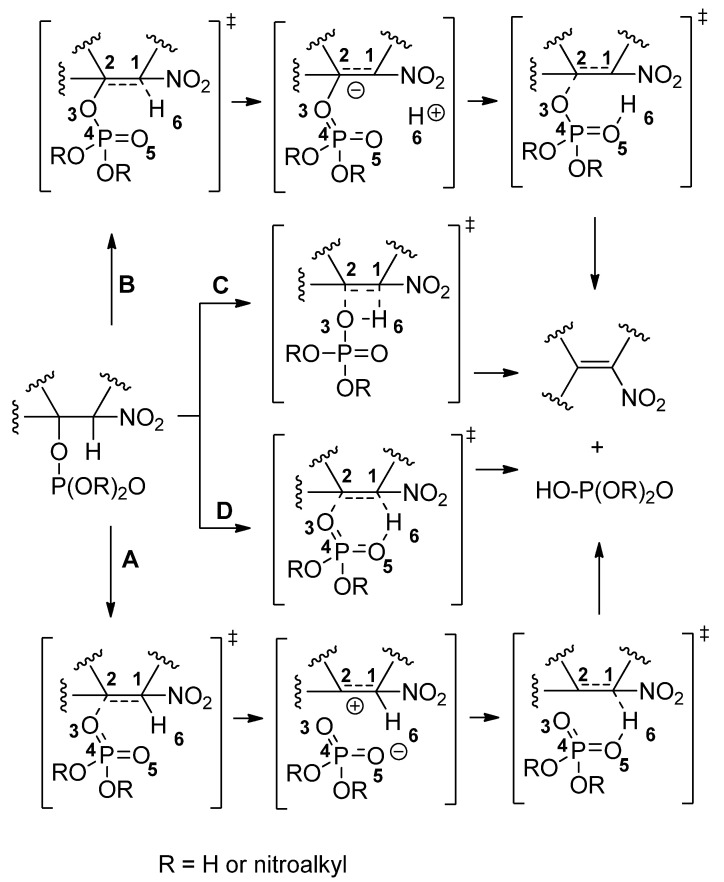
Theoretically possible mechanisms of the thermal decomposition of nitroalkyl phosphates: stepwise E1 mechanism (path **A**), stepwise E1cb mechanism (path **B**), and a single-step mechanism with pseudocyclic transition state (paths **C** and **D**).

The formation of the double bond is realized via a β-elimination scenario and is connected with the dissociation of the leaving group (Lg) as part of different types of small molecules, such as water [33,34], hydrogen chloride [35], hydrogen bromide [30], carboxylic acids [36,37,38], and phosphorous acid [39,40]. The latter group of processes is carried out at temperatures in the range of 170–185 °C and allows achieving yields of up to several dozen percent in the case of the synthesis of nitroethene [40] or, for example, 1-chloro-1-nitroethene [39]. Although the processes of dehydration, dehydrohalogenation, and carboxylic acid extrusion have been a subject of systematic mechanistic study, the process of thermal decomposition of nitroalkyl phosphates has not been explored from the mechanistic point of view. It should be underlined at this point that, in the case of the title reactions, several different types of mechanisms are possible: (i) a stepwise E1 mechanism (path **A** in Scheme 2), (ii) a stepwise E1cb mechanism (path **B** in Scheme 2), and (iii) a single-step mechanism with a pseudocyclic transition state. Next, in the last scenario, two types of eliminations can be considered: elimination via a four-membered transition state (path **C** in Scheme 2) or elimination via a six-membered transition state (path **D** in Scheme 2). Lastly, regarding many single-step processes defined earlier as “concerted”, the pericyclic mechanism has been evidently undermined in recent times [32,41,42].

**Scheme 3:**
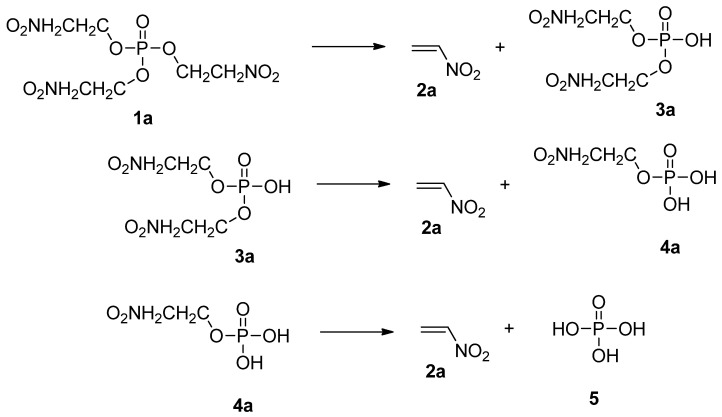
General, non-mechanistic schemes of the three-stage decomposition of tri(nitroethyl) phosphate.

So, the mechanistic aspects of the title reaction require comprehensive exploration. Within the framework of this work, we decided to shed light on the mechanism of a series of model compounds based on the results of DFT ωb97xd/6-311G(d,p) quantum-chemical calculations. For our study, we selected five model structures: simple nitroalkyl and 1- or 2-substituted by methyl or chlorine. These types of target nitroalkenes are known and have been applied in our group in organic synthesis [43,44,45,46]. Firstly, we analyzed the model processes of the decomposition of nitroalkyl ester **1**. Next, we adapted the most energetically favored scheme and explored the second and third stages of decomposition of the same ester (Scheme 3).

**Scheme 4:**
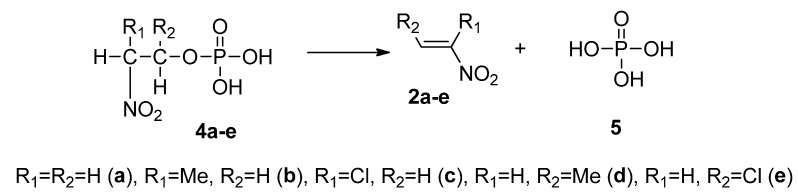
Model for the investigation of substituent effects.

Lastly, we decided to describe the effects of substituents and solvent on the reaction course (Scheme 4). These studies were supplemented using a detailed analysis of the redistribution of electron density using tools within the framework of Molecular Electron Density Theory (MEDT) [47,48]. We hope that these types of studies offer a comprehensive view of the mechanistic aspects of the title process.

## 2. Methods

### Computational Details

The exploration of the reaction profiles was performed on the basis of quantum-chemical DFT calculations. The ωB97XD/6-311G(d,p) level of theory from the *Gaussian 16* software package [49] was used. The same function was successfully used for the modeling and interpretation of many different types of pseudocyclic mechanisms, including elimination processes [50,51,52,53]. All localized and optimized stationary points have been characterized using vibrational analysis. It was found that the starting molecules, intermediates, and products had positive Hessian matrices. On the other hand, all transition states (**TS**) showed only one negative eigenvalue in their Hessian matrices. The calculations were performed at T = 297 K. Intrinsic reaction coordinate (IRC) calculations were performed for the verification of all localized transition states. The presence of a polar medium (alkyl phosphate) in the reaction environment was included using the IEFPCM algorithm [54]. Electron Localization Function (ELF) [55] analysis was implemented using the *TopMod 0.9* package [56] with a standard cubic grid step size of 0.1 Bohr. As models, the respective structures from ωB97XD/6-311G(d,p) calculations were used. To visualize the molecular geometries and ELF basin attractors, the *GaussView* 0.6 program [57] was used. The *MultiWfn 3.7* program was used for the calculation of NCI.

## 3. Results and Discussion

Our study was initiated with the exploration of the mechanism of decomposition of phosphate **1a**. We performed many tests using different types of approaches for scanning of possible changes in the molecular system. We detected that two competitive mechanisms of the transformation are possible. These are scenarios specified as **C** and **D** in Scheme 2. All attempts to localize and optimize critical structures connected with the hypothetical mechanisms **A** and **B** were not successful. So, a stepwise ionic mechanism with the participation of anionic and cationic species should be excluded for the processes analyzed.

**Figure 1:**
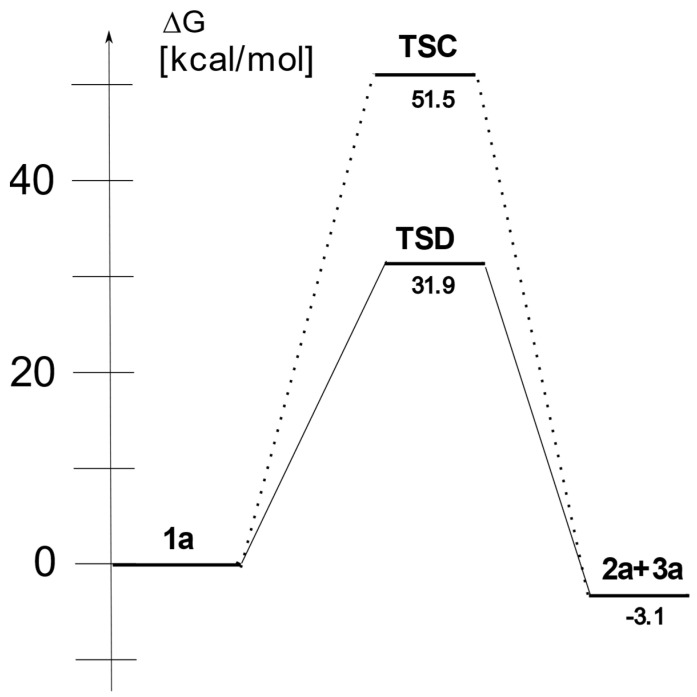
*Gibbs* free energy profiles for the competitive mechanisms of the thermal decomposition of ester **1a**, according to ωb97xd/6-311G(d,p) (PCM) calculations. The relative Gibbs free energies of transition states and products are given in kcal/mol.

**Figure 2:**
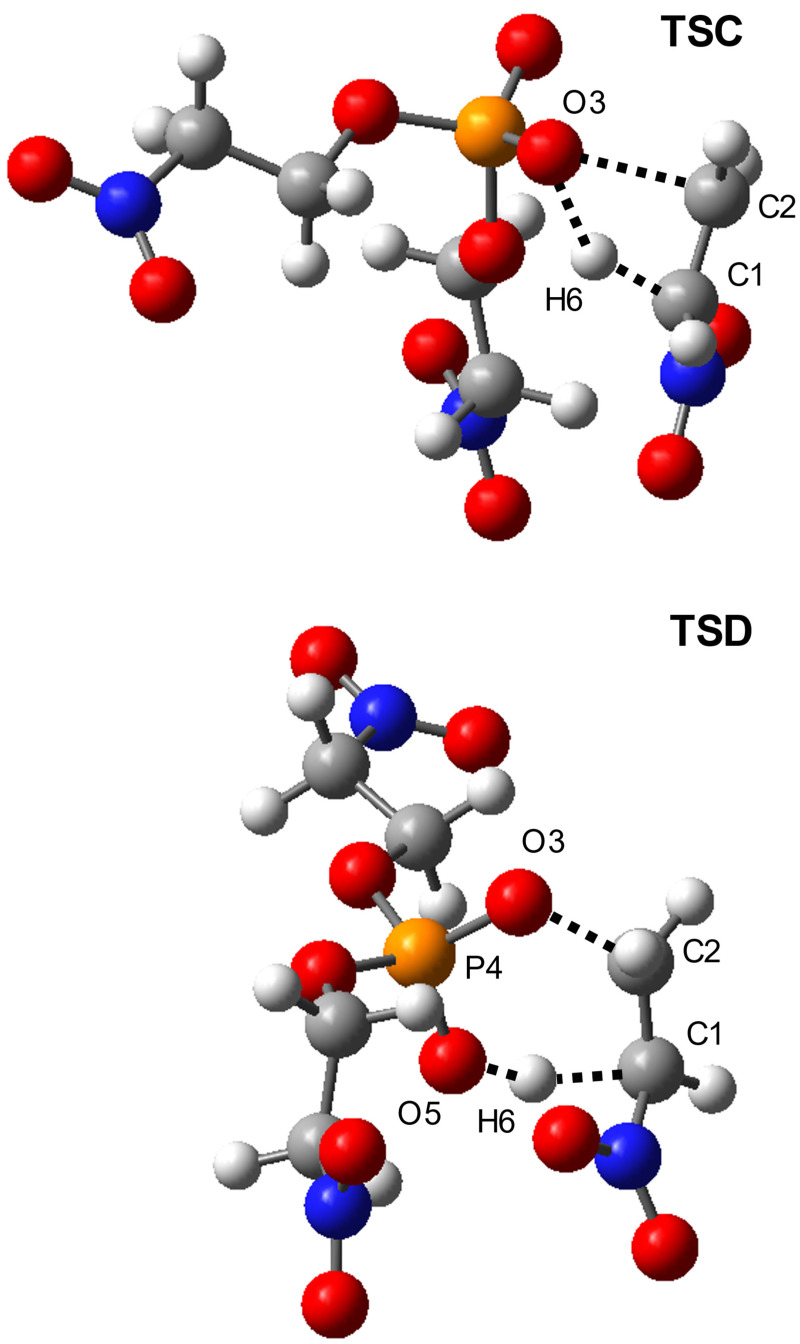
Views of transition states for the competitive mechanisms of the thermal decomposition of ester **1a**, according to ωb97xd/6-311G(d,p) (PCM) calculations.

In the case of reaction path **C**, between the valleys of the starting molecule and the products, only a single critical structure was localized (Figure 1). This is the transition state **TSC**. Achieving the area of **TSC** through the molecular system is associated with an enthalpy increase of 53.9 kcal/mol (ΔG = 51.5 kcal/mol). Including the positive entropic factors in the energy analysis results in the *Gibbs* free energy of activation being slightly lower than the enthalpy of activation. The **TSC** exhibits the nature of a four-membered cyclic structure (Figure 2). Within this molecular segment, the two σ-bonds are broken (C2-O3 and C1-H6). At the same time, a new O3-H6 σ-bond is formed, and the C1-C2 interatomic distance is slightly reduced. It should be underlined that all key distances are characterized by a typical range for C-O, C-H, and O-H bonds in transition states [50,51,58,59,60]. The IRC calculations connect the TSC structure with the valleys of the starting molecule and the products.

Alternatively, the decomposition of **1a** can be realized according to path **D**. Similarly to the case of path **C**, this reaction channel also includes only one transition state (**TSD**) (Figure 1). However, the qualitative and quantitative description of this critical point are completely different from that in the case of **TSC**. In particular, the *Gibbs* free energy of activation is only 31.9 kcal/mol. So, path **C** should be treated as the only allowed channel of the transformation of molecule **1a**. It should be emphasized that, from the energetic point of view, with the activation barrier used, the reaction should be able to proceed at a temperature of 170–185 °C, at which it actually takes place in laboratory conditions. This can be explained because, in general, six-membered transition states are formed more easily than angle-tensed four-membered transition states [61,62,63,64,65]. The reorganization of electron density is realized within the six-membered molecular segment including carbon, oxygen, phosphorus, and hydrogen atoms (Figure 2). In the framework of this structure, two σ-bonds are broken (C2-O3 and C1-H6). Atoms P4 and O5 also participate in the key segment of the transition state. Lastly, a new O5-H6 σ-bond is formed. From a structural point of view, this transition state exhibits a nature similar to respective TSs in the processes of carboxylic acid extrusion [32].

The molecular mechanisms for further decomposition of the starting molecule **1a** (**3a**→**2a** + **4a** and **4a**→**2a** + **5**) are analogous. All of these transformations are realized via the same six-membered transition state, and with a very similar barrier to the activation.

Finally, we decided to investigate the influence of the nature of the substituent and the site of substitution on the reaction course. Then, we studied the decomposition process of ester analogues **4a**, substituted at position 1 or 2 (Scheme 2) with a methyl group or a chlorine atom. It turned out that all of these transformations proceed via the same type of mechanism. All attempts to locate structures that could be associated with the hypothetical mechanisms **A** and **B** were unsuccessful. In particular, neither any ionic intermediate nor any transition states that could participate in a multi-step ionic reaction could be optimized. On the other hand, the detected mechanism via the six-membered transition state is characterized by an energy that closely matches the real reaction conditions. Therefore, this variant should be considered credible and plausible in light of the existing data.

It is important to note that the nature of the substituent influences the kinetics of the process (Table 1). The presence of a chlorine atom at position 1 determines a lower activation barrier, while a methyl group increases it. However, the same substituents at position 2 influence the reaction kinetics in exactly the opposite way. Quantitatively, the substituent effect is not strong. The described changes do not exceed 2 kcal/mol. Therefore, the reaction conditions for the basic nitroalkyl phosphates should not differ significantly from the analogous reaction involving the parent nitroethyl phosphate. The introduction of the substituent to the 4a molecule also affects the geometry of the transition state to some extent (Table S1 in the Supplementary Material). However, these changes are small and insufficient to induce a change in the transformation mechanism. Thus, the described mechanism can be considered general for a wider spectrum of nitroalkyl phosphates.

**Figure 3:**
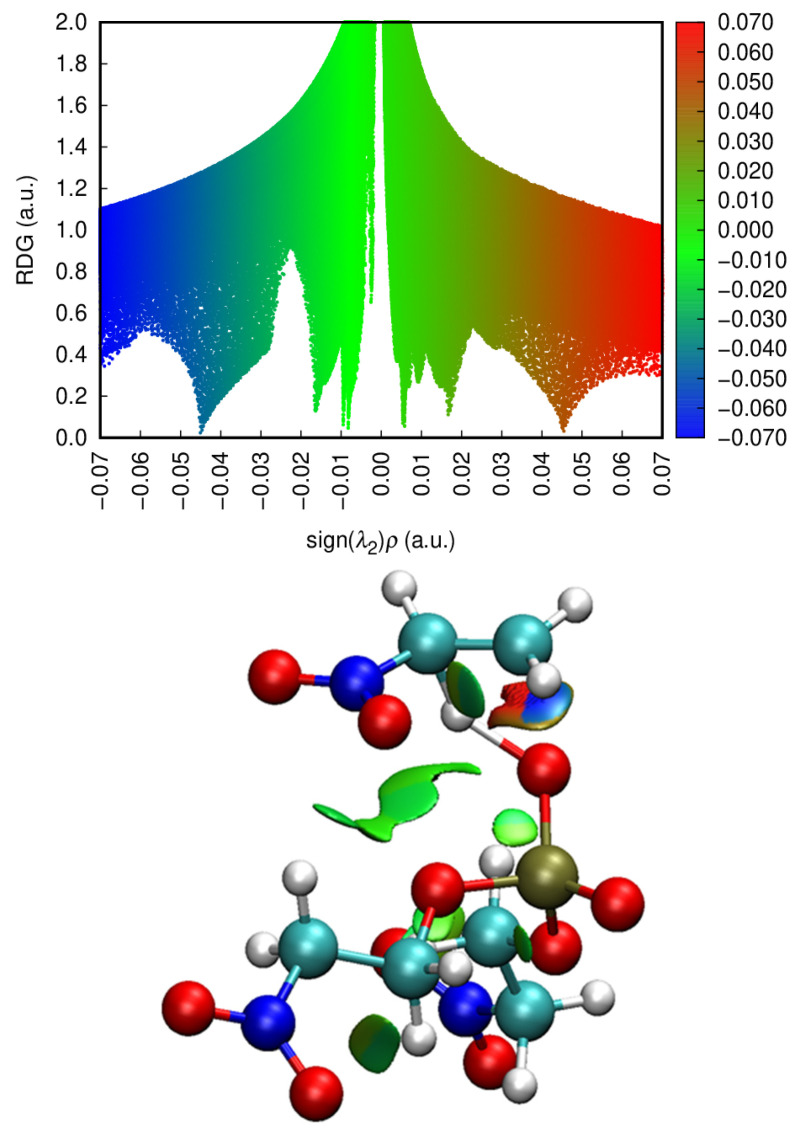
2D and 3D NCI plots for **TSC**, calculated at the ωb97xd/6-311G(d,p) (PCM) level of theory. Color coding corresponds to the range −0.07 < sign(*λ*_2_)*ρ* < 0.07 a.u.

**Figure 4:**
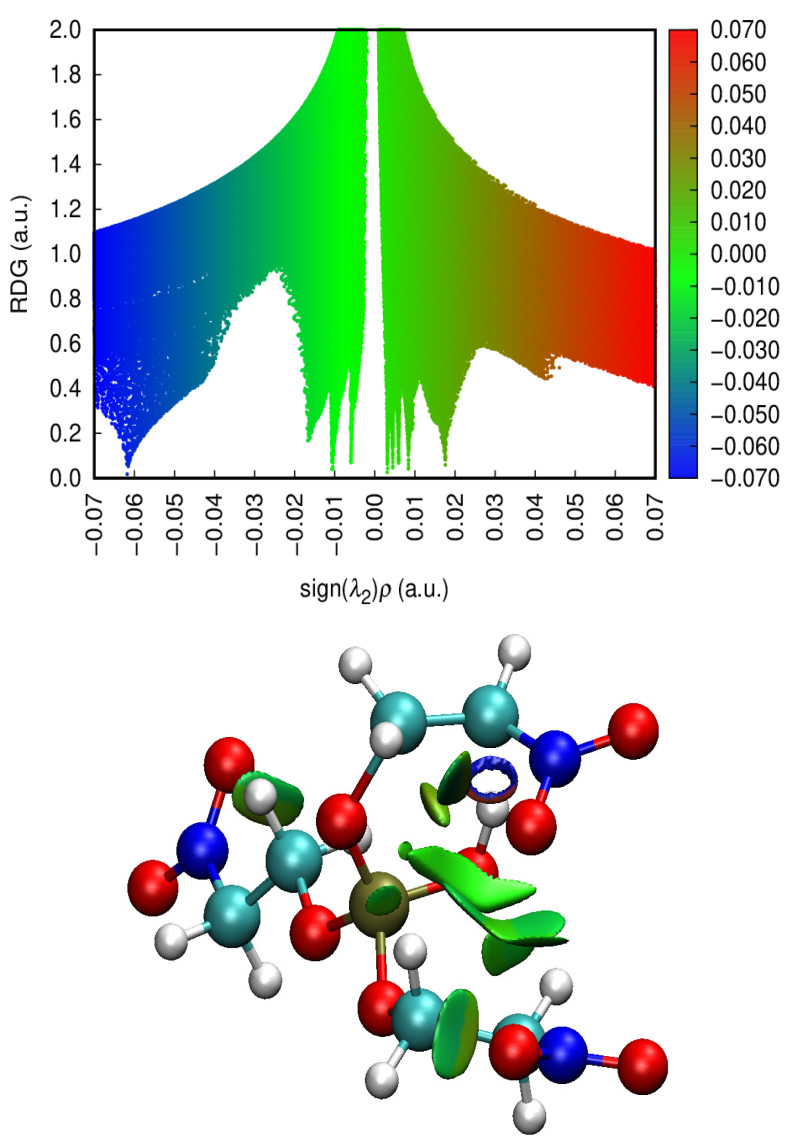
2D and 3D NCI plots for **TSD**, calculated at the ωb97xd/6-311G(d,p) (PCM) level of theory. Color coding corresponds to the range −0.07 < sign(*λ*_2_)*ρ* < 0.07 a.u.

To unveil the source of the difference in activation energies between path **C** and **D,** we performed NCI analysis [66], which allows visualization of non-covalent interactions. Figure 3 shows NCI plots for the four-membered **TSC**, where weak van der Waals stabilizing interactions (green) between the reaction center and one of the nitroethyl groups can be seen. The area where the C2-O3 bond is being broken experiences strong attraction (blue) along with steric repulsion (red) from H6, which is being abstracted from C1. In Figure 4, we can see a very different situation in the six-membered **TSD** with a much larger area of stabilizing van der Waals interactions (green) due to the less strained angles at the reaction center. Additionally, contrary to four-membered **TSC**, no steric repulsion can be seen, only a strong attraction of a hydrogen bond (blue) between H6 and the negatively charged C1.

In the next step, we analyzed the reaction mechanism of nitroethylene **2a** elimination through paths **C** and **D** using Bonding Evolution Theory (BET) analysis [67].

**Figure 5:**
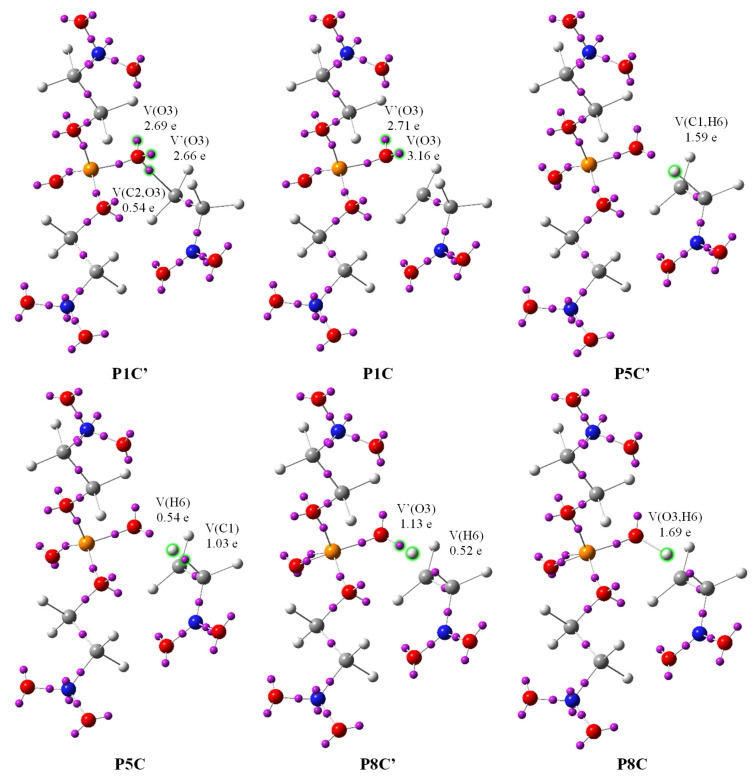
ELF valence basins attractors of structures of the most important topological changes, breaking of the C2-O3 bond and H6 migration, during the elimination reaction of nitroethane **2a** from ester **1a** through the four-membered transition state **TSC**. Structures with an apostrophe represent the first structure before the critical point.

Starting with the path leading through the four-membered transition state—Table 2 gathers the populations of the most significant valence basins at selected critical points along path **C**, and Figure 5 shows the most important structures. At the first critical point **P1C**, when d(C2,O3) = 1.960 Å, the C2-O3 bond breaks, which is embodied by the disappearance of the disynaptic basin V(C2,O3). Its population is transferred to the monosynaptic basin V(O3) (see **P1C′** and **P1C** in Figure 5). During phases II–V, the distance between C2 and O3 increases to 2.151 Å, and H6 gets closer to O3; meanwhile, electron density from the monosynaptic basins V′(O3) and V″(O3) migrates to V(O3). The system’s energy reaches its maximum of 59.4 kcal/mol at point **P3C**, which corresponds to **TSC**. Phase VI starts with the breaking of the C1-H6 bond, d(C1,H6) = 1.293 Å, and the creation of a new monosynaptic basin V(C1) with a population of 1.03 e, and a core basin V(H6) integrating 0.54 e (points **P5C′** and **P5C** in Figure 5). Next, the hydrogen atom H6 gets closer to O3; meanwhile, at point **P6C**, a new disynaptic basin V’(P4,O5) is created, decreasing the population of V(P4,O5). A new bond O3-H6, d(O3,H6) = 1.091 Å, is created by merging the monosynaptic basins V′(O3) and V(H6), integrating 1.13 e and 0.52 e, respectively (see **P8C′** and **P8C** in Figure 5). Next, at point **P9C**, a monosynaptic basin V(C1) disappears, increasing the population of the disynaptic basin V(C1,C2) to 3.48 e, which next splits into two disynaptic basins V(C1,C2) and V′(C1,C2), at point **P10C**. The remaining phases XII and XIII involves the recreation of the second monosynaptic basin V’(O3) and minor changes in the electron density of basins corresponding to the P4-O3 and P4-O5 bonds, and the monosynaptic basins of O3 and O5.

**Figure 6:**
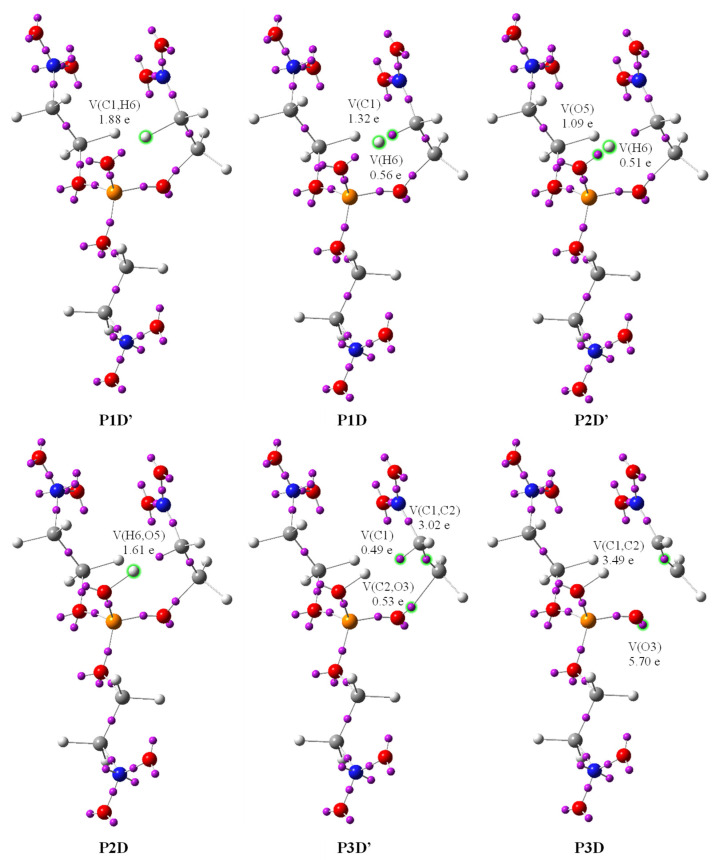
ELF valence basins attractors of structures of the most important topological changes, breaking of the C2-O3 bond and H6 migration, during the elimination reaction of nitroethane **2a** from ester **1a** through the six-membered transition state **TSD**. Structures with an apostrophe represent the first structure before the critical point.

Next, we investigated path **D** leading through the six-membered transition state. Figure 6 shows structures depicting the breaking of the C2-O3 bond and H6 migration, together with the structures directly preceding them. Table 3 collects important atom distances and populations of the most significant valence basins at the selected critical points. In contradiction to path **C**, the mechanism of path **D** starts with the breaking of C1-H6 bond, d(C1-H6) = 1.283 Å, portrayed by partitioning of the disynaptic basin V(C1,H6) into two monosynaptic basins V(C1) and V(H6) integrating 1.32 e and 0.56 e respectively (points **P1D′** and **P1D** in Figure 6). Next, phase III begins with the creation of a new bond O5-H6, d(O5-H6) = 1.074 Å, by merging the basins V′(O5) and V(H6) into a disynaptic basin V(O5,H6) with a population of 1.61 e (see **P2D′** and **P2D** in Figure 6). At the subsequent point, **P3D** breaking of bond C2-O3 takes place, d(C2-O3) = 1.949 Å, illustrated by the disappearance of the basin V(C2,O3), with its population transferred to the basin V(O3). At the same time, a monosynaptic basin V(C1) is destroyed, increasing integration of V(C1,C2) to 3.49 e (points **P3D′** and **P3D** in Figure 6). The following phase starts with the splitting of monosynaptic basin V(O5) into two basins with almost equal electron population. Next, at point **P5D**, the disynaptic basin V(C1,C2), representing the double bond C1-C2, divides into two, with the newly formed basin integrating 1.58 e. The remaining phases, VII–XI, mainly consist of electron density rearrangement on the oxygen atom O3, finally leading to two monosynaptic basins V(O3) and V′(O3).

## 4. Conclusions

Quantum chemical calculations at the ωb97xd/6-311G(d,p) level of theory (PCM) provide a clear picture of the reaction mechanism for the thermal decomposition of nitroalkyl phosphates as an effective process for obtaining various conjugated nitroalkenes. For many of them (such as 1-chloronitroethene or 1-bromonitroethene), this is the only existing synthesis pathway. Theoretical studies have demonstrated that the described processes proceed via a single-step mechanism with a six-membered transition state. However, this is not, as might seem at first glance, a “pericyclic” transition state exhibiting characteristics of an aromatic structure. BET analysis showed vastly different mechanisms for the paths leading through four- and six-membered transition states. In the first case, reaction starts with the breaking of the C2-O3 bond, followed by migration of hydrogen H6 to O3. Whereas the second one proceeds inversely, starting with the migration of H6 to O5 and subsequent breaking of the C2-O3 bond. Based on the obtained results, this mechanism can be proposed as general for a wider group of nitroalkyl phosphates.

## Figures and Tables

**Table 1:** Thermochemical parameters for the thermal decomposition of esters **4a–e** according toωb97xd/6-311G(d,p) (PCM) calculations (ΔH and ΔG in kcal/mol; ΔS in cal/molK).

Starting Ester 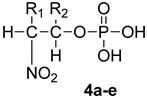			Transition	Relative Energies		
No.	R_1_	R_2_		ΔH	ΔG	ΔS
**4a**	H	H	**4a→TSD**	32.9	35.3	−8.0
			**4a→2a** + **5**	12.4	1.2	37.6
**4b**	Me	H	**4b→TSD**	36.5	37.1	−2.2
			**4b→2b** + **5**	12.3	−0.5	43.2
**4c**	Cl	H	**4c→TSD**	33.2	34.6	−4.8
			**4c→2c** + **5**	13.4	1.3	40.4
**4d**	H	Me	**4d→TSD**	32.3	33.0	−2.4
			**4d→2d** + **5**	12.4	−0.4	42.9
**4e**	H	Cl	**4e→TSD**	35.7	36.6	−2.9
			**4e→2e** + **5**	14.1	1.6	41.9

**Table 2:** ELF valence basin populations of the IRC points for nitroalkyl ester **1a**, defining the twelve different phases characterizing the thermal elimination reaction of nitroethylene **2a** and ester **3a** through the four-membered transition state. The stationary points **1a**, **TSC**, **2a**, and **3a** are also included. Distances are given in angstroms (Å), electron populations as the average number of electrons [e], relative energies in kcal/mol, and GEDT values as the average number of electrons [e].

Structures	1a	443 P1C	515 P2C	529 P3C	531 P4C	534 P5C	538 P6C	548 P7C	562 P8C	568 P9C	580 P10C	606 P11C	838 P12C	2a	3a
**Phases**	I	II	III	IV	V	VI	VII	VIII	IX	X	XI	XII	XIII		
d1(C1-H6)	1.087	1.107	1.168	1.254	1.269	1.293	1.328	1.423	1.564	1.625	1.721	1.864	2.479	-	-
d2(C2-O3)	1.435	1.960	2.137	2.149	2.151	2.153	2.155	2.159	2.166	2.171	2.192	2.254	2.821	-	-
d3(O3-H6)	2.616	2.032	1.599	1.457	1.435	1.402	1.358	1.245	1.091	1.032	0.976	0.971	0.978	-	0.963
dE	0	37.4	57.4	59.4	59.4	59.2	58.6	54.6	45.0	41.1	36.3	29.6	13.9	-	-
V(C1,C2)	1.97	2.09	2.23	2.33	2.35	2.38	2.42	2.56	2.82	3.48	1.79	1.76	1.77	1.76	-
V′(C1,C2)	-	-	-	-	-	-	-	-	-	-	1.69	1.70	1.67	1.72	-
V(C1,H6)	2.05	1.94	1.75	1.63	1.61	-	-	-	-	-	-	-	-	-	-
V(C1,N7)	2.24	2.20	2.21	2.23	2.23	2.23	2.24	2.39	2.44	2.45	2.45	2.51	2.58	2.58	-
V(C1)	-	-	-	-	-	1.03	1.05	0.95	0.68	-	-	-	-	-	-
V(C2,O3)	1.25	-	-	-	-	-	-	-	-	-	-	-	-	-	-
V(O3,H6)	-	-	-	-	-	-	-	-	1.69	1.74	1.78	1.75	1.73	-	1.74
V(O3)	2.41	3.16	2.90	3.08	4.14	4.24	4.34	4.45	4.39	4.33	4.29	2.47	2.31	-	2.24
V′(O3)	2.32	2.71	2.23	1.68	1.62	1.50	1.39	1.25	-	-	-	1.91	2.17	-	2.24
V″(O3)	-	-	0.68	1.02	-	-	-	-	-	-	-	-	-	-	-
V(P4,O3)	1.67	1.71	1.77	1.78	1.80	1.81	1.81	1.81	1.80	1.79	1.77	1.72	1.67	-	1.65
V(P4,O5)	2.07	2.05	2.05	2.05	2.05	2.05	1.21	1.18	1.27	1.27	1.30	1.35	1.43	-	1.05
V′(P4,O5)	-	-	-	-	-	-	0.79	0.83	0.81	0.82	0.80	0.77	0.69	-	0.92
V(O5)	2.97	5.82	2.00	4.23	4.18	4.12	4.03	3.78	3.67	3.63	3.66	3.61	3.69	-	3.49
V′(O5)	2.85	-	1.30	1.59	1.64	1.71	1.84	2.08	2.13	2.16	2.13	2.16	2.08	-	2.43
V″(O5)	-	-	2.52	-	-	-	-	-	-	-	-	-	-	-	-
V(H6)	-	-	-	-	-	0.54	0.47	0.43	-	-	-	-	-	-	-
V(N7)	-	0.18	0.19	0.18	0.18	0.18	0.18	-	-	-	-	0.08	-	-	-

**Table 3:** ELF valence basin populations of the IRC points for nitroalkyl ester **1a**, defining the thirteen different phases characterizing the thermal elimination reaction of the nitroethylene **2a** and ester **3a** through the six-membered transition state **TSD**. The stationary points **1a**, **TSD**, **2a,** and **3a** are also included. Distances are given in angstroms (Å), electron populations as the average number of electrons [e], relative energies in kcal/mol, and GEDT values as the average number of electrons [e].

Structures	1a	153 P1D	168 P2D	209 P3D	211 P4D	223 P5D	239 P6D	251 P7D	254 P8D	255 P9D	259 P10D	2a	3a
**Phases**	I	II	III	IV	V	VI	VII	VIII	IX	X	XI		
d1(C1-H6)	1.087	1.283	1.628	1.937	1.948	2.012	2.098	2.166	2.183	2.189	2.212	-	-
d2(C2-O3)	1.435	1.587	1.636	1.949	1.965	2.057	2.171	2.250	2.269	2.275	2.300	-	-
d3(O5-H6)	2.745	1.372	1.074	0.987	0.985	0.982	0.980	0.980	0.980	0.980	0.980	-	0.962
dE	0.6	25.9	34.7	29.9	29.4	26.4	22.8	20.5	20.0	19.9	19.3	-	-
V(C1,C2)	2.00	2.08	2.24	3.49	3.49	1.90	1.87	1.84	1.83	1.83	1.82	1.76	-
V′(C1,C2)	-	-	-	-	-	1.58	1.6	1.63	1.64	1.64	1.65	1.72	-
V(C1,H6)	2.04	-	-	-	-	-	-	-	-	-	-	-	-
V(C1,N7)	2.23	2.48	2.62	2.58	2.57	2.54	2.51	2.49	2.49	2.49	2.49	2.58	-
V(C1)	-	1.32	1.23	-	-	-	-	-	-	-	-	-	-
V(C2,O3)	1.28	1.05	0.99	-	-	-	-	-	-	-	-	-	-
V(O3)	4.69	4.83	4.84	5.70	5.70	5.74	1.79	2.06	1.85	2.14	3.55	-	3.20
V′(O3)	-	-	-	-	-	-	3.99	1.27	1.32	1.35	2.24	-	2.69
V″(O3)	-	-	-	-	-	-	-	2.45	2.32	2.30	-	-	-
V‴(O3)	-	-	-	-	-	-	-	-	0.30	-	-	-	-
V(P4,O3)	1.69	1.77	1.83	2.03	2.04	2.04	2.04	2.05	2.04	2.05	2.04	-	1.99
V(P4,O5)	2.07	1.92	1.82	1.72	1.7	1.69	1.67	1.66	1.66	1.66	1.65	-	1.65
V(O5)	2.97	4.90	4.54	4.46	2.36	2.34	2.25	2.19	2.17	2.17	2.15	-	2.44
V′(O5)	2.83	0.91	-	-	2.13	2.15	2.24	2.31	2.32	2.32	2.35	-	2.05
V(O5,H6)	-	-	1.61	1.72	1.72	1.72	1.73	1.73	1.73	1.73	1.73	-	1.73
V(H6)	-	0.56	-	-	-	-	-	-	-	-	-	-	-

## Data Availability

The original contributions presented in this study are included in the article/Supplementary Material. Further inquiries can be directed to the corresponding author.

## Supplementary Materials

The following supporting information can be downloaded at https://www.mdpi.com/article/10.3390/ma18235312/s1, Table S1: Key parameters for critical structures of the thermal decomposition of esters **4a**–**e** according to ωb97xd/6-311G(d,p) (PCM) calculations. Views of representative TSs are presented in Figure 2.
