# Supplementary material for: A Mechanistic Insight into the Molecular Mechanism of the Thermal Decomposition of Nitroalkyl Phosphates: MEDT Computational Study
Source: Materials (Basel). 2025 Nov 25;18(23):5312. doi: 10.3390/ma18235312 (PMC12692962; doi:10.3390/ma18235312)
Supplement: Supplementary file 1 [file materials-18-05312-s001.zip › materials-3956874-supplementary.pdf]

SUPPLEMANTARY MATERIAL

A mechanistic insight on the molecular mechanism of the thermal decomposition of nitroalkyl phosphates: MEDT computational study

Przemysław Woliński [1], Ewa Dresler [2] and Radomir Jasiński [1,*]

[1] Cracow University of Technology, Department of Organic Chemistry and Technology, Warszawska 24, 31-155 Kraków, Poland; przemyslaw.wolinski@pk.edu.pl; radomir.jasinski@pk.edu.pl

[2] Łukasiewicz Research Network—Institute of Heavy Organic Synthesis "Blachownia", Energetyków 9, 47-225 Kędzierzyn-Koźle, Poland; ewa.dresler@icso.lukasiewicz.gov.pl

* Correspondence: radomir.jasinski@pk.edu.pl

Table S1. Key parameters for critical structures of the thermal decomposition of esters **4a-e** according to the ωb97xd/6-311G(d,p) (PCM) calculations. Views of representative TSs are presented on the Figure 2.

**Starting ester** 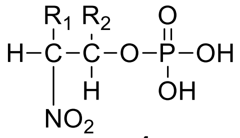 **4a-e**			**Reaction**	**Structure**	**Interatomic distances**					
Nr	R_1	R_2			C1-C2	C2-O3	O3-P4	P4-O5	O5-H6	H6-C1
**4a**	H	H	**4a→2a+5**	**4a**	1.521	1.433	1.594	1.471	2.662	
				**TSD**	1.432	1.676	1.521	1.554	1.034	1.730
				**2a**	1.320					
				**5**			1.467	1.592	0.961	
**4b**	Me	H	**4b→2b+5**	**4b**	1.518	1.427	1.596	1.472	3.351	
				**TSD**	1.439	1.673	1.522	1.553	1.037	1.709
				**2b**	1.324					
**4c**	Cl	H	**4c→2c+5**	**4c**	1.531	1.420	1.601	1.473	2.371	
				**TSD**	1.424	1.674	1.520	1.566	0.988	1.999
				**2c**	1.320					
**4d**	H	Me	**4d→2d+5**	**4d**	1.519	1.443	1.590	1.472	3.262	
				**TSD**	1.437	1.736	1.519	1.548	1.072	1.602
				**2d**	1.326					
**4e**	H	Cl	**4e→2e+5**	**4e**	1.519	1.393	1.615	1.470	2.328	
				**TSD**	1.427	1.681	1.524	1.557	1.016	1.796
				**2e**	1.324					
